# Meta-transcriptomic analysis reveals a new subtype of genotype 3 avian hepatitis E virus in chicken flocks with high mortality in Guangdong, China

**DOI:** 10.1186/s12917-019-1884-y

**Published:** 2019-05-06

**Authors:** Xue-Lian Zhang, Wen-Feng Li, Sheng Yuan, Jin-Yue Guo, Zhi-Li Li, Shi-Hong Chi, Wen-Jing Huang, Xiao-Wen Li, Shu-Jian Huang, Jian-Wei Shao

**Affiliations:** 1 Key Laboratory for Preventive Research of Emerging Animal Diseases, Foshan University, Foshan, 528231 Guangdong China; 2 College of Life Science and Engineering, Foshan University, Foshan, 528231 Guangdong China; 3 Department of Medical Microbiology and Parasitology, Zhejiang University School of Medicine, Hangzhou, 310058 Zhejiang China

**Keywords:** Avian hepatitis E virus, High mortality, Meta-transcriptomics analysis, New subtype

## Abstract

**Background:**

Hepatitis E virus (HEV) is one of most important zoonotic viruses, and it can infect a wide range of host species. Avian HEV has been identified as the aetiological agent of big liver and spleen disease or hepatitis-splenomegaly syndrome in chickens. HEV infection is common among chicken flocks in China, and there are currently no practical measures for preventing the spread of the disease. The predominant avian HEV genotype circulating in China have been identified as genotype 3 strains, although some novel genotypes have also been identified from chicken flocks in China.

**Results:**

In this study, we used a meta-transcriptomics approach to identify a new subtype of genotype 3 avian HEV in broiler chickens at a poultry farm located in Shenzhen, Guangdong Province, China. The complete genome sequence of the avian HEV, designated CaHEV-GDSZ01, is 6655-nt long, including a 5′ UTR of 24 nt and a 3′ UTR of 125 nt (excluding the poly(A) tail), and contains three open reading frames (ORFs). Sequence analysis indicated that the complete ORF1 (4599 nt/1532 aa), ORF2 (1821 nt/606 aa) and ORF3 (264 nt/87 aa) of CaHEV-GDSZ01 share the highest nucleotide sequence identity (85.8, 86.7 and 95.8%, respectively) with the corresponding ORFs of genotype 3 avian HEV. Phylogenetic analyses further demonstrated that the avian HEV identified in this study is a new subtype of genotype 3 avian HEV.

**Conclusions:**

Our results demonstrate that a new subtype of genotype 3 avian HEV is endemic in Guangdong, China, and could cause high mortality in infected chickens. This study also provides full genomic data for better understanding the evolutionary relationships of avian HEV circulating in China. Altogether, the results presented in this study suggest that more attention should be paid to avian HEV and its potential disease manifestation.

## Background

Hepatitis E virus (HEV) is the causative agent of hepatitis E, which is an important public health concern in many parts of the world, particularly in developing countries [1, 2]. HEV is a non-enveloped, single-stranded positive-sense RNA virus that belongs to the family Hepeviridae and includes two genera, *Orthohepevirus* and *Piscihepevirus* [3]. The genome encodes three open reading frames (ORFs) flanked by a capped 5′ terminus and a polyadenylated 3′ terminus. Among the three ORFs, ORF1 is the longest and is located at the 5′ terminus of the genome; this ORF encodes a non-structural polyprotein including a methyltransferase, a papain-like cysteine protease, a viral helicase, and an RNA-dependent RNA polymerase (RdRp) [4]. ORF2 is located at the 3′ end of the genome and encodes a capsid protein, which is the major structural protein and functionally binds to host cells, induces neutralizing antibody production, and participates in viral particle assembly [5, 6]. ORF3, which overlaps with ORF2, encodes a cytoskeleton-associated phosphoprotein that interacts with the ORF2 protein and a number of cellular signal transduction pathway proteins [7, 8].

As an important zoonotic virus, HEV can infect a wide range of host species, and its host spectrum is ever expanding. Since the first animal strain of HEV (i.e., swine HEV) was isolated and characterized in 1997 in the United States [9], numerous strains of HEV have been isolated from a number of different animal species and a wide range of geographic locations. Countries that have reported HEV infections include Japan, Taiwan, New Zealand, mainland China, South Korea, Hungary, Australia, the United Arab Emirates, and the Netherlands, among others. The infected animal species include domestic and wild pigs, chickens, rabbits, ruminants, ferrets, minks, bats, rodents, and aquatic birds [3, 10].

Based on sequence analysis, most of the HEVs identified thus far belong to the genus *Orthohepevirus,* which contains four species, designated A, B, C, and D [11, 12]. Avian HEV, belonging to *Orthohepevirus* B, has been identified as the aetiological agent of big liver and spleen disease (BLSD) or hepatitis-splenomegaly syndrome (HS syndrome) in broiler breeder hens and laying hens aged 30 to 72 weeks, via the faecal-oral route [13]. Avian HEV infection can cause increased mortality, a reduction in egg production or subclinical infection, resulting in large economic losses in the poultry industry [14, 15]. The avian HEV genome shares approximately 50% nucleotide sequence identity and some similar antigenic epitopes with mammalian HEVs [13]. Various avian HEVs have been classified into four different genotypes based on full or nearly complete genomes: genotype 1, which is mainly found in Australia and Korea [16, 17], genotype 2, from the USA and Korea [13, 18], genotype 3, from Europe and China [19, 20], and genotype 4, from Hungary and Taiwan [21, 22].

Avian HEV genotype 3 was first characterized in Hungary in 2005 and was later detected in the United Kingdom, Germany, and Austria before 2007 [16]. In China, an Avian HEV genotype 3 strain designated CaHEV was first detected and characterized in Shandong Province in 2010 [20]. Since then, avian HEV strains genotype 3 have been identified in chickens with HS syndrome in many provinces of China [15, 23–26]. In recent years, many previously undescribed genotypes of avian HEV have been found in different regions of China [27, 28], which suggests that there is much greater diversity of avian HEV circulating in chicken flocks in China than previously indicated.

## Results

### Meta-transcriptomics based pathogen discovery

Through an unbiased high-throughput RNA sequencing approach, a total of 42,682,590 paired-end sequencing reads were generated, resulting in 14.5 GB of fastq format sequence data. After default quality control (QC) and de-barcoding steps provided by the Illumina platform, 40,531,484 reads (95%) remained for further analysis.

**Fig. 1:**
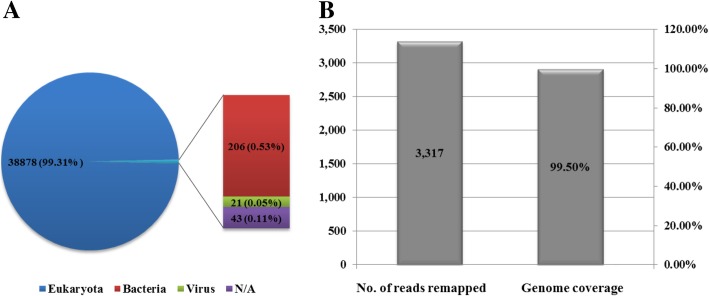
(**a**) Homology search results for contigs assembled from NGS clean reads . (**b**) Total NGS clean reads remapped to the reference sequence and genome coverage

After de novo assembly using Trinity, a total of 48,891 contigs were generated, which varied from 201 to 9584 nt in length. These contigs were compared to non-redundant nucleotide databases (nt) via nucleotide Blast searches. A total of 38,878 contigs (79.1%), 206 contigs (0.53%), and 21 contigs (0.05%) were annotated as belonging to eukaryotes, bacteria, and viruses, respectively. The remaining 43 contigs (0.11%) were labelled as N/A, showing no taxonomic information (Fig. 1a). All the contigs annotated as eukaryotic were identified as coming from chicken RNA sources, and no fungal pathogens were found. Those contigs identified as bacterial came from species from the genera *Escherichia*, *Acinetobacter*, *Bradyrhizobium*, *Bacillus*, and *Rhizobacter*, which are ubiquitous in the natural environment. Those contigs identified as viral were annotated as avian HEV and avian leukosis virus (ALV). Strikingly, the assembled contigs covered the nearly complete genome sequence of avian HEV. The sequences were confirmed by reverse transcription polymerase chain reaction (RT-PCR), and 5′ and 3′ RACE were used to obtain the terminal sequences. The virus sequence obtained in this study was designated CaHEV-GDSZ01 and has been deposited in GenBank under accession number MK050107.

The sequence reads were subsequently mapped to the virus genome to estimate sequencing coverage and depth. Using the complete virus genome of CaHEV-GDSZ01 as the reference sequence, 3317 reads were remapped to the avian HEV sequence, with 99.5% genome coverage (6621/6655 nt) and pairwise identity of 97.1% at a mean depth of 8× (Fig. 1b).

### Sequence comparison

The complete genome of CaHEV-GDSZ01 consisted of 6655 nucleotides (nt) excluding the poly(A) tail at the 3′ end, and it contained three major ORFs. The genome comprised a 5′ UTR of 24 nt (1–24), ORF-1 of 4599 nt (25–4623), encoding a non-structural polyprotein of 1532 amino acids, ORF-2 of 1821 nt (4710–6530), encoding a capsid protein of 606 amino acids, ORF-3 of 264 nt (4657–4920), encoding a cytoskeleton-associated phosphoprotein of 87 amino acids, and a 3′ UTR of 125 nt (6531–6655).

Multiple sequence comparisons based on individual ORFs showed that the complete ORF1 of CaHEV-GDSZ01 shared 80.8 to 85.8% nucleotide sequence identity and 92.0 to 95.0% amino acid sequence identity with reference strains. The nucleotide sequence identity between the complete ORF2 of CaHEV-GDSZ01 and the reference strains varied from 83.5 to 86.7%, while the amino acid sequence identity between them was 98.0 to 99.3%, which were higher identities than those of the complete ORF1 (Table 1). Additionally, sequence comparison of the complete ORF3 with reference strains revealed that they shared 92.8–95.8% nucleotide sequence identity and 89.7–100% amino acid sequence identity, which was significantly higher than the identities of ORF1 and ORF2. Additionally, the nucleotide sequence identities of the 5’UTR and 3’UTR of CaHEV-GDSZ01 were 64.0–88.0% and 54.3–83.7%, respectively, compared to other avian HEV strains (Table 2).

**Table 1:** Comparison of the nucleotide and amino acid identities of complete ORF1and ORF2 gene sequences among avian HEV strains

Gene	Strains	1	2	3	4	5	6	7	8	9	10	11	12
ORF1	1 MK050107/China		81.2	80.8	80.8	85.6	85.8	82.6	83.3	81.1	81.5	81.5	81.6
	2 AM943647/ Australia	**92.2**		86.5	86.3	81.5	81.7	80.2	81.0	81.3	81.5	81.5	81.9
	3 JN597006/South Korea	**92.4**	**95.9**		91.5	81.8	81.7	81.0	80.9	81.5	81.6	81.6	81.9
	4 KC454286/South Korea	**92.0**	**96.1**	**97.8**		81.0	81.1	80.2	81.2	81.2	81.5	81.5	81.4
	5 GU954430/China	**94.8**	**93.0**	**92.6**	**92.2**		98.3	82.5	83.1	81.1	80.6	80.6	81.6
	6 AM943646/Hungary	**94.8**	**93.1**	**93.0**	**92.6**	**98.8**		83.0	83.3	81.2	80.9	80.9	81.6
	7 JN997392/Hungary	**93.7**	**92.0**	**92.2**	**91.8**	**94.1**	**94.3**		87.3	80.5	81.1	81.1	81.1
	8 KF511797/Taiwan	**95.0**	**93.1**	**93.3**	**92.8**	**94.8**	**94.9**	**95.9**		81.6	81.4	81.4	81.6
	9 KM377618/South Korea	**92.3**	**93.3**	**93.0**	**92.8**	**93.0**	**93.2**	**92.5**	**93.1**		89.8	89.8	89.3
	10 AY535004/United States	**92.6**	**93.7**	**93.4**	**93.3**	**93.0**	**93.2**	**92.7**	**93.6**	**96.9**		100	89.6
	11 NC023425/ United States	**92.6**	**93.7**	**93.4**	**93.3**	**93.0**	**93.2**	**92.7**	**93.6**	**96.9**	**100**		89.6
	12 EF206691/ United States	**93.2**	**93.9**	**93.5**	**93.5**	**92.8**	**93.0**	**92.6**	**93.4**	**96.5**	**97.4**	**97.4**	
ORF2	1 MK050107/China		84.8	83.5	84.0	86.7	86.5	86.7	84.9	83.9	84.5	84.5	84.7
	2 AM943647/ Australia	**98.3**		87.9	88.1	84.4	84.1	83.9	84.1	83.6	84.3	84.3	84.5
	3 JN597006/South Korea	**98.2**	**98.8**		91.6	85.0	85.0	84.2	84.4	85.0	86.2	86.2	84.6
	4 KC454286/South Korea	**98.0**	**98.3**	**98.3**		84.8	84.6	84.7	84.5	83.8	84.5	84.5	84.2
	5 GU954430/China	**99.0**	**98.8**	**98.7**	**98.5**		98.5	87.3	85.0	84.0	84.5	84.5	84.1
	6 AM943646/Hungary	**98.7**	**98.7**	**98.3**	**98.2**	**99.7**		87.1	84.8	83.6	84.4	84.4	84.0
	7 JN997392/Hungary	**99.3**	**98.7**	**98.5**	**98.3**	**99.7**	**99.3**		87.9	84.0	84.9	84.9	84.6
	8 KF511797/Taiwan	**98.5**	**98.7**	**98.2**	**98.0**	**99.2**	**99.0**	**99.0**		84.6	84.3	84.3	84.7
	9 KM377618/South Korea	**97.9**	**98.2**	**98.0**	**97.9**	**98.3**	**98.2**	**98.2**	**98.5**		91.7	91.7	89.6
	10 AY535004/USA	**98.5**	**98.8**	**99.0**	**98.2**	**99.0**	**98.7**	**98.8**	**98.5**	**98.7**		100	90.7
	11 NC023425/USA	**98.5**	**98.8**	**99.0**	**98.2**	**99.0**	**98.7**	**98.8**	**98.5**	**98.7**	**100**		90.7
	12 EF206691/USA	**98.2**	**98.5**	**98.3**	**97.5**	**98.3**	**98.2**	**98.2**	**98.5**	**98.7**	**99.0**	**99.0**	

The comparison was done using MegAlign ClustalW analysis. Boldface indicates percentage identities of amino acid sequences

**Table 2:** Comparison of the nucleotide and/or amino acid sequences identities of complete ORF3, 5’UTR and 3’UTR gene sequences among avian HEV strains

Gene	Strains	1	2	3	4	5	6	7	8	9	10	11	12
ORF3	1 MK050107/China		93.9	92.8	92.8	95.5	95.8	95.8	95.8	93.2	93.2	93.2	93.2
	2 AM943647/ Australia	**95.4**		96.6	95.8	93.9	93.6	94.3	94.3	95.5	95.5	95.5	95.5
	3 JN597006/South Korea	**94.3**	**96.6**		95.8	93.6	93.2	93.9	93.2	95.1	95.1	95.1	95.1
	4 KC454286/South Korea	**93.1**	**95.4**	**96.6**		93.6	93.2	93.9	94.3	93.9	93.2	93.2	93.2
	5 GU954430/China	**94.3**	**92.0**	**90.8**	**92.0**		98.9	95.8	95.1	93.2	93.9	93.9	93.9
	6 AM943646/Hungary	**94.3**	**92.0**	**90.8**	**92.0**	**97.7**		96.2	95.5	92.8	93.6	93.6	93.6
	7 JN997392/Hungary	**96.6**	**94.3**	**93.1**	**92.0**	**93.1**	**93.1**		97.0	92.8	92.8	92.8	94.3
	8 KF511797/Taiwan	**100**	**95.4**	**94.3**	**93.1**	**94.3**	**94.3**	**96.6**		92.0	92.0	92.0	92.0
	9 KM377618/South Korea	**92.0**	**95.4**	**92.0**	**90.8**	**87.4**	**87.4**	**89.7**	**92.0**		97.0	97.0	97.0
	10 AY535004/United States	**93.1**	**96.6**	**93.1**	**92.0**	**88.5**	**88.5**	**90.8**	**93.1**	**98.9**		100	97.0
	11 NC023425/ United States	**93.1**	**96.6**	**93.1**	**92.0**	**88.5**	**88.5**	**90.8**	**93.1**	**98.9**	**100**		97.0
	12 EF206691/ United States	**89.7**	**94.3**	**90.8**	**89.7**	**88.5**	**88.5**	**93.1**	**89.7**	**94.3**	**95.4**	**95.4**	
3’UTR /5’UTR	1 MK050107/China		77.5	76.7	74.4	82.9	83.7	54.3	74.4	78.3	76.0	76.0	81.4
	2 AM943647/ Australia	—		89.1	89.9	79.1	80.6	55.0	76.0	79.1	79.8	79.8	78.3
	3 JN597006/South Korea	*84.0*	—		96.1	81.4	82.2	53.5	82.9	77.5	77.5	77.5	76.7
	4 KC454286/South Korea	*88.0*	—	*96.0*		79.1	79.8	52.7	79.1	78.3	76.7	76.7	76.0
	5 GU954430/China	*64.0*	—	*64.0*	*68.0*		97.7	54.3	83.7	85.3	83.7	83.7	85.3
	6 AM943646/Hungary	**—**	—	—	—	—		55.8	81.4	86.0	82.9	82.9	86.0
	7 JN997392/Hungary	—	—	—	—	—	—		51.2	55.8	53.5	53.5	55.8
	8 KF511797/Taiwan	*80.0*	—	*88.0*	*92.0*	*60.0*	—	—		79.8	84.5	84.5	82.2
	9 KM377618/South Korea	*88.0*	—	*96.0*	*100*	*68.0*	—	—	*92.0*		89.1	89.1	93.0
	10 AY535004/USA	*88.0*	—	*96.0*	*100*	*68.0*	—	—	*92.0*	*100*		100.0	91.5
	11 NC023425/USA	*88.0*	—	*96.0*	*100*	*68.0*	—	—	*92.0*	*100*	*100*		91.5
	12 EF206691/USA	*88.0*	—	*96.0*	*100*	*68.0*	—	—	*92.0*	*100*	*100*	*100*	

The comparison was done using MegAlign ClustalW analysis. Boldface indicates percentage identities of amino acid sequences. The italic font numbers show the nucleotide sequences identities of 5’UTR among the avian HEV strains. —: Sequence couldn’t be obtained from GenBank

### Phylogenetic analysis

To examine the evolutionary relationships of avian HEV determined in this study with other virus strains, phylogenetic analyses were performed based on the complete nucleotide sequences of ORF1 and ORF2 of HEV. All the phylogenetic trees were estimated using the maximum likelihood method with 1000 bootstrap replicates, and the cutthroat trout virus was used as the outgroup in each case.

**Fig. 2:**
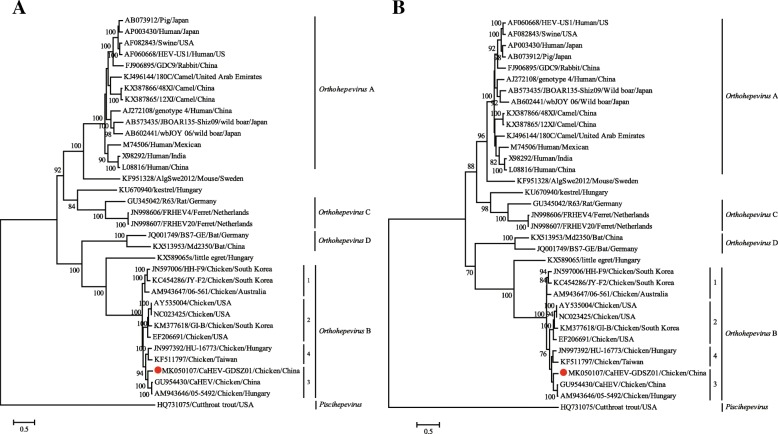
Phylogenetic analysis based on the complete nucleotide sequences of ORF1 (**a**) and ORF2 (**b**) and reference isolates. The trees were constructed based on the maximum likelihood method implemented in PhyML v3.0. Bootstrap values were calculated with 1000 replicates of the alignment. GenBank accession numbers are followed by the name of avian HEV strains. Red dots indicate the avian HEV determined in this study

Phylogenetic analysis based on the complete ORF1 showed that all known Orthohepeviruses, including the virus newly identified CaHEV-GDSZ01, were divided into four clades. All the viruses isolated in chickens, including CaHEV-GDSZ01, clustered together and constituted the species *Orthohepevirus* B. Furthermore, CaHEV-GDSZ01 was clustered together with two avian HEVs isolated in China and Hungary with short branch lengths and was allocated to the genotype-3 subclade of the *Orthohepevirus* B species (Fig. 2a). A similar phylogenetic relationship was observed in a maximum-likelihood tree based on the complete sequences of ORF2 (Fig. 2b).

### Selection pressure analysis

**Fig. 3:**
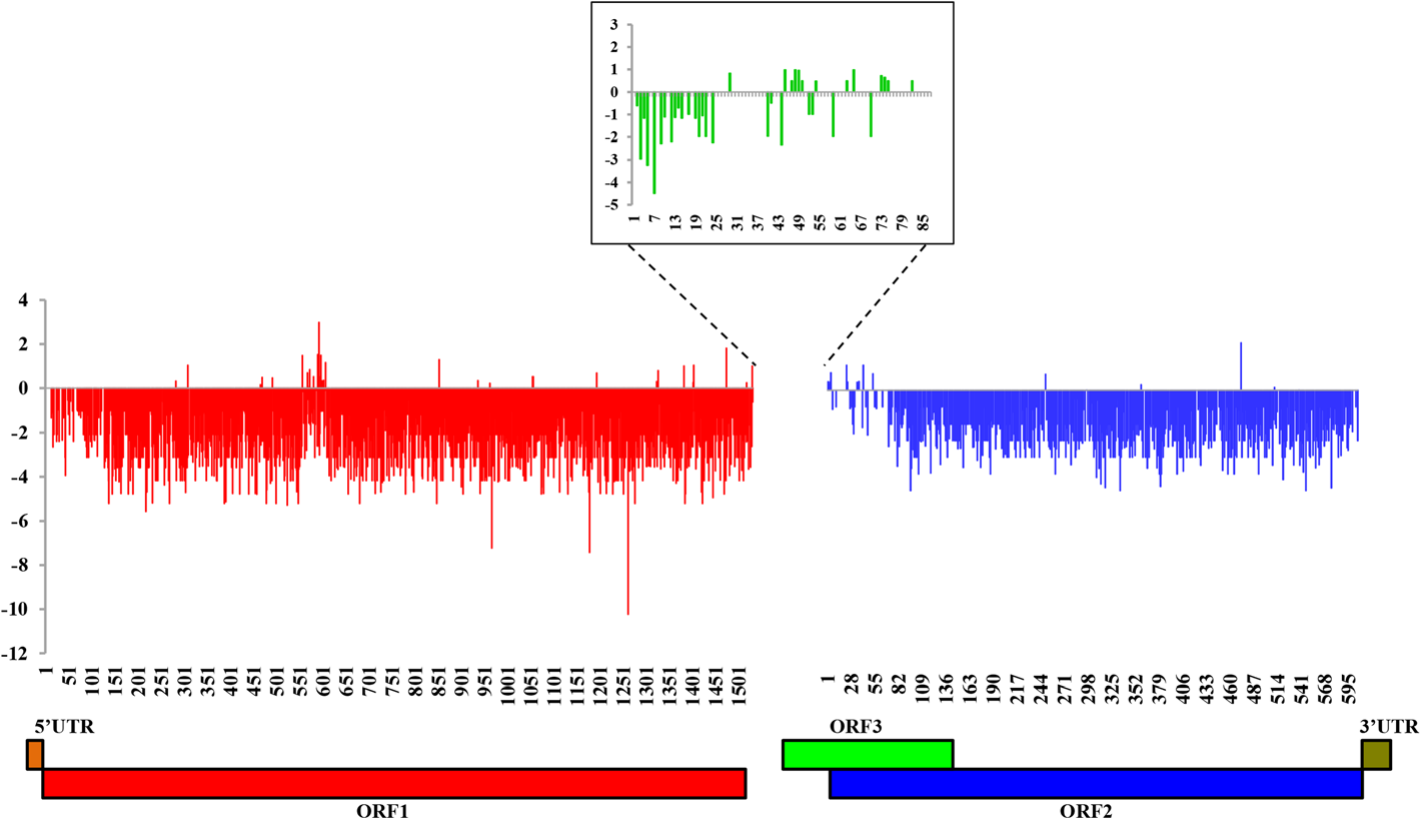
Differences between non-synonymous and synonymous (d*N*-d*S*) rates plotted for avian HEV ORF1, ORF2 and ORF3. d*N*-d*S* < 0 indicates a negatively selected site. d*N*-d*S* > 0 indicates a positively selected site

In the selection pressure analyses, as shown in Fig. 3, the mean d*N*-d*S* values of ORF1, ORF2, and ORF3 were − 2.04, − 1.61, and − 0.37, respectively. Many negatively selected sites were observed in ORF1, ORF2, and ORF3 (642, 305, and 7, respectively), while no positively selected sites were found, which suggests a lack of positive selected sites in the three ORFs examined.

## Discussion

Avian HEV causes diseases that threaten the healthy development of the poultry industry worldwide. Since the CaHEV strain was first detected and characterized in China in 2010, avian HEV genotype 3 strain has been identified as prevalent in many regions of China [15, 23–26]. In recent years, many new avian HEV strains belonging to a tentatively novel genotype of species *Orthohepevirus* B were detected in chickens in Hebei, Guangdong, Anhui, and Jilin Provinces, China [28]. In this study, a new subtype of genotype 3 avian HEV strain was determined in broiler chickens with high mortality showing the clinical symptoms of HS syndrome through unbiased high-throughput sequencing. All of these results suggest that there is a larger diversity of avian HEV circulating in China, and avian HEV infection should be monitored as an emerging disease agent on chicken farms.

The comparative analysis of individual ORFs among avian HEV strains, including CaHEV-GDSZ01, showed 80.8–85.8%, 83.5–86.7%, and 92.8–95.8% nucleotide sequence identities for ORF1, ORF2, and ORF3, respectively. Comparison of the amino acid sequence showed higher identities: 92.0–95.0% for ORF1, encoding a non-structural polyprotein; 98.0%-99.3 for ORF2, encoding a capsid protein; and 89.7–100% for ORF3, encoding a cytoskeleton-associated phosphoprotein. However, the nucleotide sequence identities of ORF3 among all the avian HEV strains were often higher than the amino acid sequence identities, suggesting that non-synonymous mutations may occur more frequently in this fragment (Table 2).

In the phylogenetic analysis of the complete ORF1 and ORF2, CaHEV-GDSZ01 was grouped with other viruses of the *Orthohepevirus* B species isolated from chickens, which were clustered into the genotype-3 subclade with two avian HEVs isolated in China and Hungary. These results were consistent with the results of sequence comparison analyses of ORF1 and ORF2 indicating that CaHEV-GDSZ01 showed the highest nucleotide sequence identity with the isolate from Hungary (accession no. AM943646; 85.8% identity) and the isolate from China (accession no. GU954430; 86.7% identity). Taken together, these results suggest that CaHEV-GDSZ01 may be a new subtype of avian HEV genotype 3.

In selection pressure analyses, negatively selected sites were observed predominantly in ORF1, ORF2, and ORF3, and no positively selected sites were observed, suggesting that negative selection was predominant in ORF1, ORF2, and ORF3. Additionally, the mean d*N*-d*S* values of the three ORFs were − 2.04, − 1.61, and − 0.37, respectively, also reflecting the predominance of negative selection. Therefore, the results showed that the microevolution of avian HEV seems to be driven by negative selection (d*N* < d*S*) of all the three ORFs (summarized in Fig. 3). This conclusion is consistent with the expected behaviour of a small-genome virus, in which most components are probably essential for viral viability.

## Conclusion

Through unbiased high-throughput sequencing, we identified a new subtype of genotype 3 avian HEV from a poultry farm experiencing high mortality of broiler chickens showing HS syndrome. In combination with previous studies, our results suggest a larger diversity of avian HEV circulating in China, and much greater efforts should be exerted towards the surveillance of avian HEV.

## Methods

### Case history and clinical sample collection

In May 2018, many 130-day-old broiler chickens on a poultry farm in Shenzhen, Guangdong Province, China, experienced a sudden mass die-off. The species of these chickens was Qingyuan partridge chicken, one of the three prevalent chicken species in Guangdong Province, and all the chickens were are free roaming. All the deceased chickens died naturally, and the clinical symptoms and postmortem lesions presented by the affected chickens were recorded. The deceased chickens were subjected to necropsy, and tissue samples from heart, liver, spleen, lung, kidney, duodenum, brain, pancreas, and bursa of fabricius were aseptically collected and snap-frozen in liquid nitrogen immediately and stored at − 80 °C for further use.

### Total RNA extraction, library construction and NGS sequencing

Approximately 100 mg of liver tissue from a chicken with hepatitis-splenomegaly syndrome was homogenized in 600 μL of lysis buffer by using a TissueRuptor instrument (Qiagen). Total RNA was extracted by using an RNeasy Plus Minikit according to the manufacturer’s instructions. The quantity and quality of extracted RNA was evaluated with a NanoDrop 2000 (Thermo Fisher Scientific, Waltham, USA).

Ten RNA samples of liver tissues were pooled as one mixed sample in equal-mass amounts. RNA library preparation was conducted following the methodology previously described by Pettersson et al. [29]. Briefly, the host ribosomal RNA (rRNA) was depleted by using a Ribo-Zero-Gold (Epidemiology) kit (Illumina Inc., USA). Subsequently, a library was constructed based on the rRNA-depleted RNA samples using a TruSeq total RNA library preparation kit (Illumina). Library cDNA levels were quantified before, during and after library preparation with Qubit (ThermoFisher Scientific) high-sensitivity RNA/DNA assays, and the fragment sizes were checked with a Bioanalzyer (Agilent Technologies). Paired-end (150-bp) sequencing was then performed on the Illumina Hiseq2500 platform.

### Transcriptome assembly and contig annotation

Transcriptome analyses were conducted following the methodology previously described by Shi et al. [30]. Sequencing reads were demultiplexed and trimmed for quality with Trimmomatic [31] before de novo assembly using Trinity [32]. The resulting contigs were compared against the entire nonredundant protein (nr) database downloaded from GenBank by using BLASTX with an E-value cutoff of 1E-5. Unmatched sequence reads were assembled using Trinity. To confirm the assembly results, the reads were mapped back to the target contigs with Bowtie2 [33], and any assembly errors were inspected by using Integrated Genomics Viewer (IGV) [34]. The final sequences of the virus genomes were obtained from the majority consensus of the mapping assembly.

### Sequence confirmation by RT-PCR and 5′/3’RACE

To confirm the sequences of the virus genomes and fill the gaps generated in the merging of viral contigs with unassembled overlaps by using the SeqMan program implemented in the Lasergene software package v7.1 (DNASTAR), reverse transcriptase-PCR was conducted. All primers were designed based on the transcriptome assembly sequence, and amplicons were sequenced directly by Sanger sequencing. The 5′ and 3′ ends of the genome of avian HEV determined in this study were obtained by 5′ and 3′ rapid amplification of cDNA ends (RACE) using a RACE kit (TaKaRa, China). All primer sequences are available upon request. Sequences were assembled and manually edited to produce the final viral genomes using the SeqMan program (DNASTAR, Madison, WI).

### Sequence comparison and phylogenetic analysis

DNASTAR’s Lasergene 12 Core Suite was used for Sanger sequencing assembly and nucleotide sequence translation. Sequence similarity was evaluated via a BLASTn search in GenBank (http://blast.ncbi.nlm.nih.gov/Blast.cgi). The alignment of the sequences obtained in this study and the existing reference sequences in GenBank was carried out using ClustalW (default parameters) as implemented in the MEGA program, version 6.0 [35].

The phylogenetic trees were estimated following the methodology previously described by Lu et al. [36]. The best-fit evolutionary model for all sequence alignments was determined using jModel Test version [37]. The General Time Reversible (GTR) nucleotide substitution model with a gamma (Γ)-distribution model of among-site rate variation and a proportion of invariable sites (i.e. GTR + Γ + I) were found to be the best-fit model for these sequences. Phylogenetic trees were estimated using the maximum-likelihood (ML) method with bootstrap support values calculated from 1000 replicates implemented in PhyML v3.0 [38]. All phylogenetic trees were mid-point rooted for purposes of clarity only.

### Selection pressure analyses

The selection pressure of code sites was examined site by site across the entire coding region of the genome using the DataMonkey (http://www.datamonkey.org) web server and assessed by calculating the difference between non-synonymous (d*N*) and synonymous (d*S*) rates (d*N* − d*S*) for each ORF. All the analyses of the three ORFs were performed using the SLAC method and the REV nucleotide substitution bias model, and a *P*-value < 0.1 indicated negatively selected sites.

## Abbreviations

BLSD: Big liver and spleen disease
HEV: Hepatitis E virus
HS syndrome: Hepatitis-splenomegaly syndrome
ORF: Open reading frame
RdRp: RNA-dependent RNA polymerase
rRNA: Ribosomal RNA
RT-PCR: Reverse transcription-polymerase chain reaction
UTR: Untranslated region
